# The impact of jawbone regions (molar area, premolar area, anterior area) and bone density on the accuracy of robot-assisted dental implantation: a preliminary study

**DOI:** 10.3389/fbioe.2025.1536957

**Published:** 2025-02-25

**Authors:** Mirealimu Miadili, Xiaoman Li, Yan Zhang, Danping Ruan, Wei Liu, Jianfei Zhang, Yiming Gao

**Affiliations:** ^1^ Shanghai Jiao Tong University School of Medicine, Institute of Ruijin Hospital, Shanghai, China; ^2^ Department of Oral and Cranio-maxillofacial Surgery, Shanghai Ninth People’s Hospital, Shanghai Jiao Tong University School of Medicine, Shanghai, China; ^3^ Department of Stomatology, Minhang Central Hospital Fudan University, Shanghai, China; ^4^ The Fourth Affiliated Hospital of Inner Mongolia Medical University, Baotou, China

**Keywords:** robot-assisted, implantation, accuracy, bone density, jawbone

## Abstract

Robotic-assisted dental implantation represents a transformative innovation in modern dentistry, offering enhanced surgical precision and reduced variability. Despite its clinical adoption, the impact of anatomical and bone-related factors on placement accuracy remains underexplored. This retrospective study evaluated 54 implants placed in 30 patients using cone-beam computed tomography (CBCT) and virtual planning software to analyze deviations in crown position, apex position, and angulation. Significant regional variations in accuracy were observed, with higher angular deviations in the anterior maxilla (mean ± SD: 3.21° ± 2.22°) and greater positional deviations in the posterior mandible (1.09 mm ± 0.51 mm) (*p* < 0.05). Implant diameter significantly influenced global deviation (*p* = 0.019), while implant length and bone density (classified by Misch’s system) showed no significant effects (*p* > 0.05). However, denser bone types (D1) exhibited a trend toward increased deviations, potentially due to insertion resistance. These findings underscore the need for region-specific and bone-quality considerations in robotic-assisted implantation. Refining robotic navigation and feedback mechanisms is critical to optimizing accuracy, particularly in anatomically complex regions.

## Introduction

With continuous advancements in technology, robotic-assisted dental implantation has emerged as a promising approach to enhance surgical precision, stability, and reduce trauma (Block and Emery, 2016). Compared to freehand procedures, robotic systems have demonstrated significantly improved accuracy in implant placement. However, current evidence suggests that this precision may vary depending on anatomical regions—such as the anterior, premolar, and molar areas—and differences in bone density. These variations could be attributed to factors such as anatomical structure, cortical bone thickness, and operational angulation, which can influence implant positioning (Wu et al., 2024). Despite these potential challenges, limited studies have systematically investigated how these site-specific characteristics impact the accuracy of robotic-assisted implantation (Jorba-García et al., 2021).

In conventional implant surgery, the precision of implant positioning largely relies on the clinician’s skill and experience, leading to variability across complex anatomical regions (D'Haese et al., 2017). Robotic systems theoretically mitigate these challenges by offering advanced navigation and real-time feedback, yet achieving uniform accuracy across all jawbone areas remains uncertain (Wang M. et al., 2024). This uncertainty is particularly pronounced in regions with dense cortical bone, thinner bone structures, or steep angulations, which may contribute to positional deviations during implant placement (Howashi et al., 2016).

Currently, clinical investigations on the relationship between bone density and robotic-assisted implant precision remain scarce. While imaging modalities such as Cone Beam Computed Tomography (CBCT) provide valuable diagnostic data, they lack the resolution to accurately quantify bone density (Visconti et al., 2013). To address this limitation, bone classification systems such as Misch’s system are widely employed to categorize bone quality (Misch, 1990). Nevertheless, significant differences in density and bone types across jaw regions may still impact implant accuracy, requiring further exploration (Putra et al., 2020).

This study aims to investigate how specific jawbone regions—namely the anterior, premolar, and molar areas—affect the precision of robotic-assisted dental implantation. By examining these regional variations, this study seeks to provide insights that can guide clinical decision-making and optimize implant outcomes across diverse anatomical scenarios.

## Materials and methods

### Study design and patient selection

This retrospective study included a cohort of 30 patients who underwent robot-assisted dental implant surgeries at RuiJin Hospital from 1 January 2022 to 31 December 2023. The study included 19 males and 11 females, aged 22–75 years, with an average age of 43 years, comprised a total of 54 implant placements, each of which was performed using a robot-assisted surgical system specifically designed for dental implantology (Table 1). The inclusion criteria for patient selection were as follows: (1) patients over the age of 18, (2) requiring one or more dental implants, (3) without significant systemic health conditions that could affect bone metabolism (e.g., osteoporosis or uncontrolled diabetes), and (4) having sufficient bone volume for implant placement without the need for advanced grafting procedures. Patients with craniofacial deformities, history of radiation therapy to the head or neck, or those on medications affecting bone density (e.g., bisphosphonates) were excluded. All patients provided informed consent, and the study protocol was approved by the institutional review board (IRB) of Ruijin Hospital Ethics Committee, Shanghai Jiaotong University School of Medicine.

**TABLE 1:** Demographic and clinical characteristics of the included patients.

Personal details		
Age	22–75	43
Gender	Male	19
	Female	11
Implant position		
Maxillary	Anterior	10
	Premolar	9
	Molar	9
Mandibular	Anterior	9
	Premolar	8
	Molar	9
Implant system	Straumann	54
Implant diameter	3.3	24
	4.1	18
	4.8	12
Implant length	8	7
	10	19
	12	28
Bone density	D1	9
	D2	23
	D3	13
	D4	9

### Sample size calculation

The sample size for this study was determined using an evidence-based design approach. *A priori* statistical power analysis was performed using G*Power 3.1 (Heinrich-Heine University Düsseldorf, Germany) with a significance level (α) of 0.05 and statistical power (1−β) of 0.80. Based on previous studies on dental implants, a large effect size (Cohen’s d = 0.8) was anticipated, and a two-tailed independent samples t-test calculated the minimum required sample size for each group to be 25. Additionally, the sample size was adjusted considering the following factors: to ensure clinical independence, an average of 1–2 implants per patient was considered a reasonable protocol; the number of eligible patients at the center over the past 2 years ensured practical feasibility; and the sample size was designed to ensure representativeness and generalizability of the results. This approach balanced statistical rigor with clinical and practical considerations.

Ultimately, 30 patients and 54 implants (28 maxillary and 26 mandibular) were included in the study, exceeding the minimum sample size required for adequate statistical power (25 per group). While this sample size was sufficient to detect large effect size differences, subgroup analyses by implant position (e.g., maxillary anterior: 10 implants, maxillary premolar: 9 implants, maxillary molar: 9 implants; mandibular anterior: 9 implants, mandibular premolar: 8 implants, mandibular molar: 9 implants) may have limited statistical power. These subgroup analyses should therefore be interpreted as exploratory.

### Preoperative assessment and surgical planning

**FIGURE 1:**
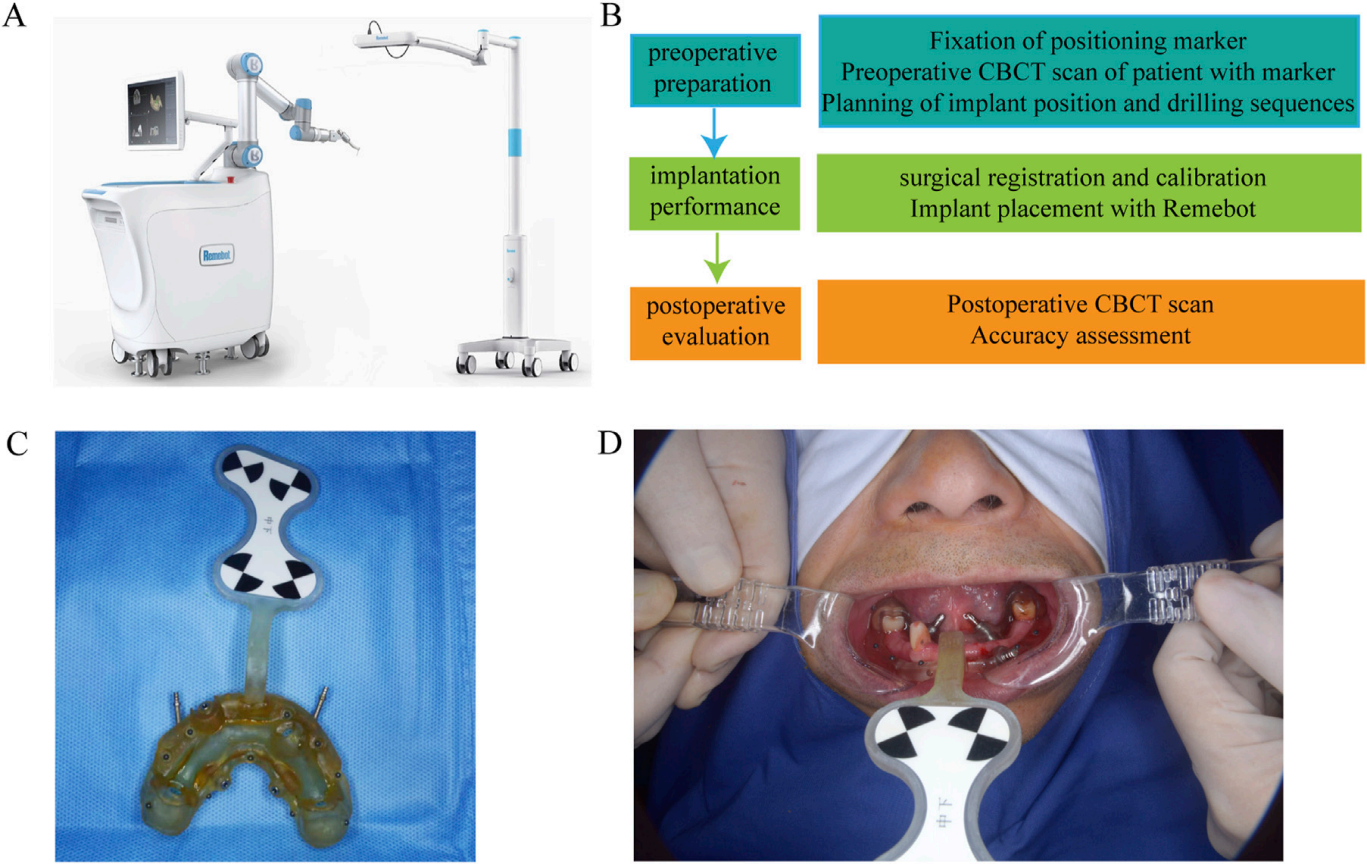
**(A)** RemebotDent1.0 navigation system. **(B)** Preoperative Assessment and Surgical Planning. **(C)** A printed prosthesis with marked ceramic beads. **(D)** Install the prosthesis and match the implantation plan file.

Prior to surgery, all patients underwent detailed preoperative assessments using cone-beam computed tomography (CBCT) scans to evaluate the morphology and density of the jawbone at the planned implant sites. All CBCT scans were acquired using standardized parameters, including tube voltage (90.0 kV), tube current (6.0 mA), and resolution (0.500 × 0.500 voxel), ensuring consistency and comparability of imaging data. The CBCT data was imported into specialized three-dimensional (3D) planning software (RemebotDent1.0 navigation system), which allowed for precise virtual surgical planning. Since the Hounsfield Unit (HU) values derived from Cone-Beam Computed Tomography (CBCT) do not directly correlate with the density of the jawbone, an alternative and indirect method was employed to address this limitation (Hu et al., 2024) (Figures 1A–D). Specifically, we utilized Misch’s jawbone classification system to categorize implant sites, and the detailed classification methodology is elaborated in the subsequent section on classification. By adopting this method, we aimed to achieve a more reliable evaluation of the bone quality at the implant site, ensuring a robust basis for clinical decision-making. All the implant sites were catalogued into three anatomical regions: the anterior region (incisors and canines), premolar region, and molar region.

For each implant, the optimal positioning, angulation, and depth were planned virtually, with the goal of ensuring proper primary stability and avoiding critical anatomical structures such as the inferior alveolar nerve, maxillary sinus, and adjacent tooth roots. The planned implant positions served as the reference standard against which the actual postoperative positions would be compared (de Almeida et al., 2010). To ensure consistency and minimize operator-dependent variability, all preoperative planning and surgical procedures were performed by the same experienced clinician. This approach maximized the elimination of potential operator-related errors, ensuring the accuracy and reproducibility of the study results.

### Surgical procedure

**FIGURE 2:**
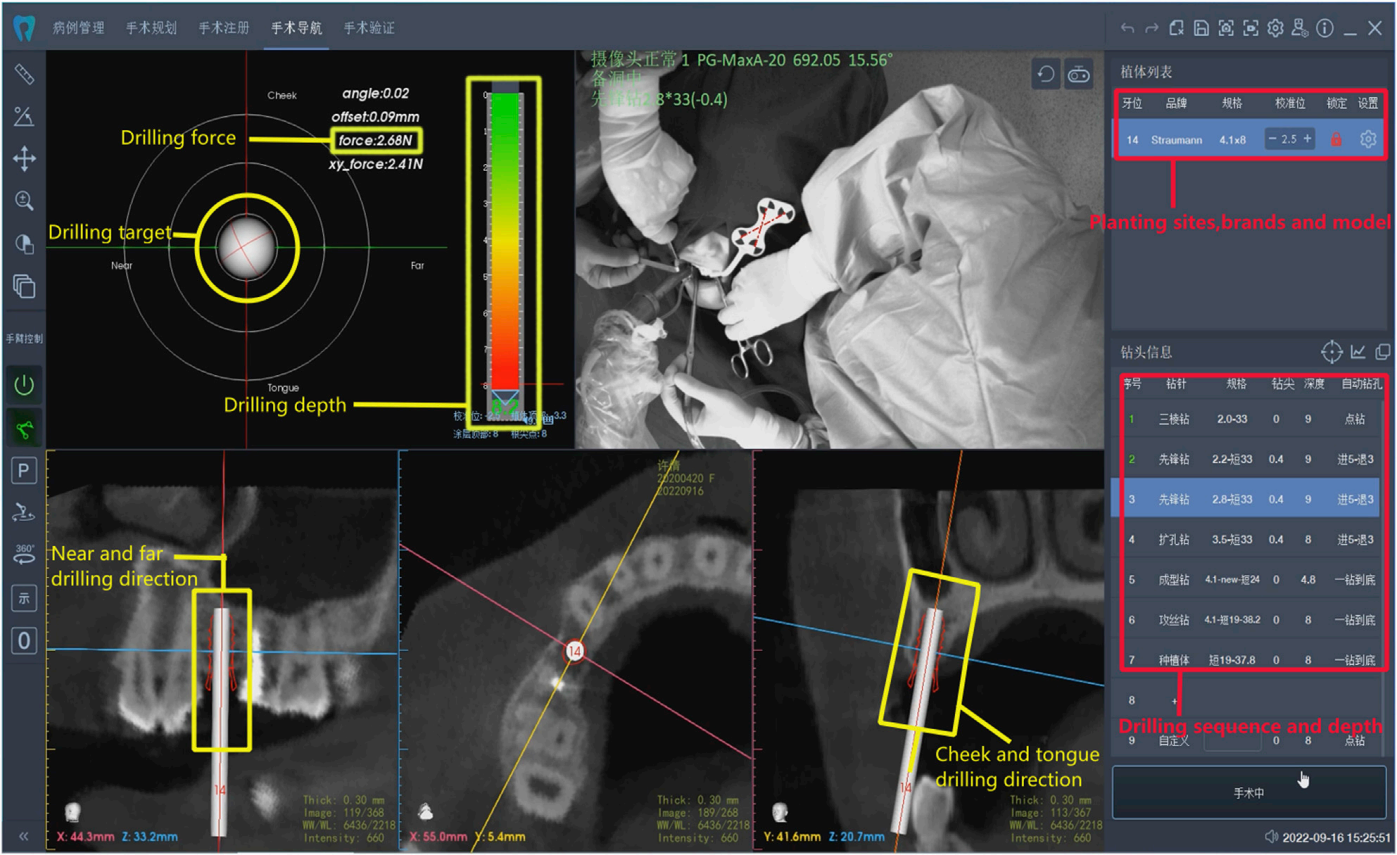
Robotic system that provides real-time feedback and automated controls.

Robot-assisted dental implant surgeries were performed using the Remebot, (Beijing Baihui Weikang Technology Co., Ltd., Beijing, China),a precision-guided robotic system that provides real-time feedback and automated controls to enhance the accuracy of implant placement. Following the preoperative planning, the surgical robot was calibrated to execute the implant placement according to the 3D virtual plan (Figure 2). The robot-guided system allowed for controlled drilling and precise insertion of the implants. All procedures were performed by experienced surgeons with expertise in robot-assisted dental surgery, ensuring consistency across the patient cohort (Bahrami et al., 2024).

### Postoperative evaluation

Postoperative CBCT scans were performed immediately after surgery to assess the actual implant positions. Using the same 3D software, the postoperative implant positions were compared with the preoperative virtual plan to measure deviations. Deviations were measured in three primary domains: (1) Crown Position Deviation: Measured in both lateral (buccolingual/mesiodistal) and depth (vertical) dimensions at the level of the implant crown. (2) Apex Position Deviation: Similarly, deviations in the apex (root) position were measured in lateral and depth dimensions. (3) Angulation Deviation: The angular deviation was measured as the difference between the planned and actual angulation of the implant in degrees (Takács et al., 2023).

**FIGURE 3:**
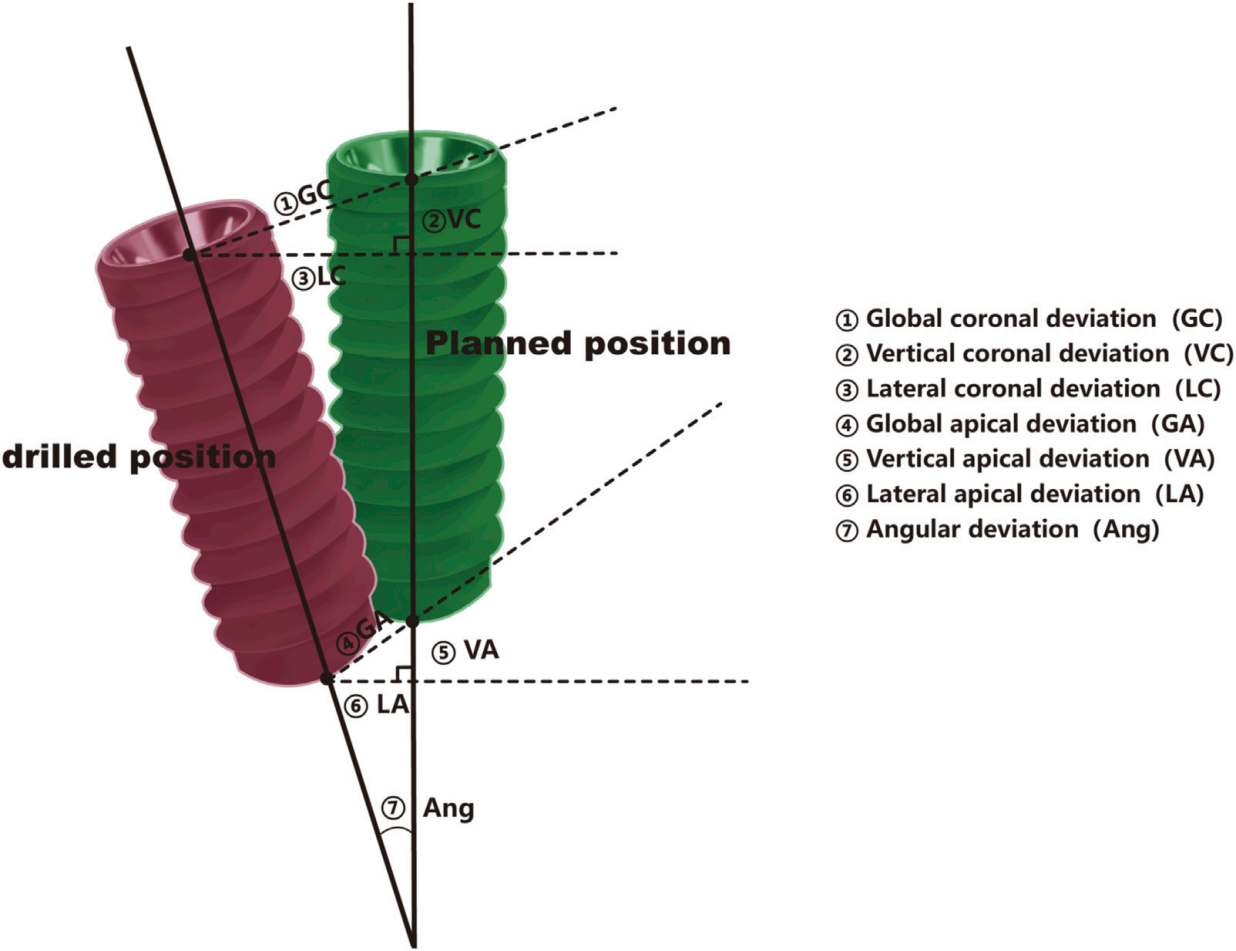
Pattern diagram, the pattern is divided into planned position (green)and drilling position (red).

**FIGURE 4:**
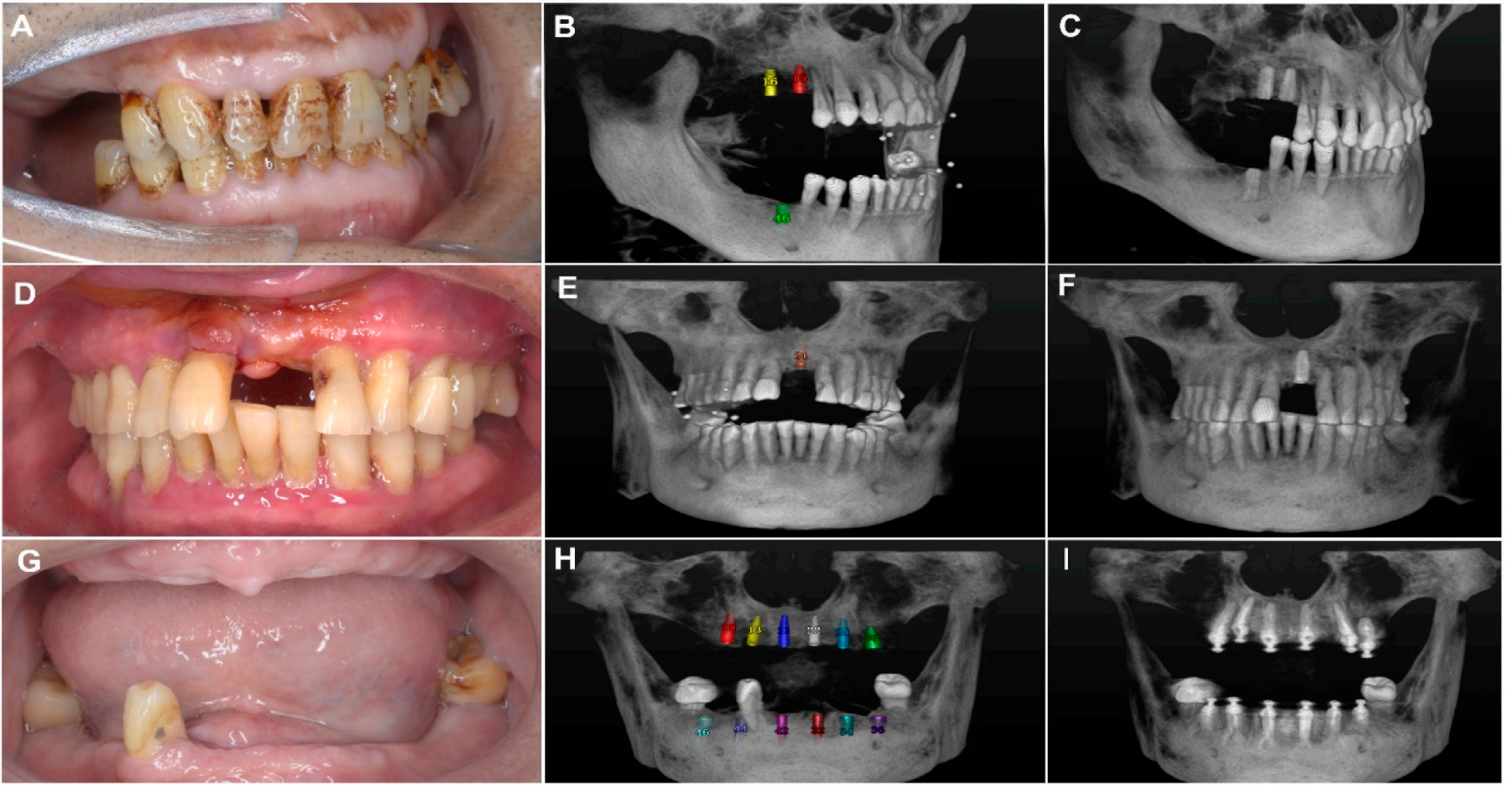
**(A)** Intraoral photo of the patient showing missing premolars and molars. **(B)** Three-dimensional imaging showing simulated implant placement at the edentulous sites with ceramic bead markers. **(C)** Postoperative 3D view showing the actual positions of the implants. **(D)** Intraoral photo of the patient showing missing maxillary anterior teeth. **(E)** 3D imaging illustrating the simulated implant placement at the edentulous sites in the maxillary anterior region with ceramic bead markers. **(F)** Postoperative view showing the actual implant positions. **(G)** Intraoral photo of the patient showing edentulism (complete tooth loss). **(H)** 3D imaging showing simulated implant placement, including the planned positions of multiple implants. **(I)** Postoperative 3D view showing the reconstructed dental arch.

Each deviation (Figure 3) was precisely calculated using the digital measurement tools provided by the 3D software, ensuring high accuracy and reproducibility of measurements (Figures 4A–I). The results were recorded in millimeters (mm) for positional deviations and degrees (°) for angular deviations (Shi et al., 2024).

### Bone density analysis using Misch’s classification

Bone density was assessed using Misch’s classification system, which categorizes bone into four types based on cortical and trabecular characteristics. CBCT imaging served as the primary diagnostic tool, providing detailed visualization of the maxilla and mandible. Experienced clinicians reviewed the CBCT scans, incorporating their expertise to classify bone quality into Type I (dense cortical bone), Type II (dense cortical and trabecular bone), Type III (thin cortical with dense trabecular bone), or Type IV (thin cortical and sparse trabecular bone). All classifications were documented systematically for subsequent analysis. This approach integrates advanced imaging with expert interpretation to enhance the precision of bone density evaluation (Misch, 1989).

### Statistical analysis

All data were analyzed using SPSS 20.0 statistical software (IBM Corp., Armonk, NY, USA). Descriptive statistics, including mean values, standard deviations, and 95% confidence intervals (CI), were calculated for all measured deviations (crown, apex, and angulation). One-way analysis of variance (ANOVA) was employed to evaluate discrepancies in implant accuracy under various conditions, including maxillary and mandibular implant accuracy, regional implant accuracy within the maxilla and mandible (categorized into anterior, premolar, and molar regions), implant length, implant diameter, and bone density. Jawbone density was classified according to Misch’s classification system, and ANOVA was used to determine whether bone density significantly influenced implant accuracy. A trend indicating higher deviations in denser bone types was observed and further assessed. To control for the accumulation of Type I error due to multiple comparisons, Bonferroni corrections were applied. Specifically, for comparisons involving six tooth positions, three implant diameters, three implant lengths, and four bone density categories, the adjusted significance thresholds were α' = 0.008, 0.017, 0.017, and 0.0125, respectively. An independent samples t-test was used to compare maxillary and mandibular groups, with a significance level of α = 0.05 and *p*-values derived from two-tailed tests. Effect sizes were calculated using Cohen’s d and classified as follows: d < 0.2 (small effect size), 0.2 ≤ d < 0.5 (small to medium effect size), 0.5 ≤ d < 0.8 (medium effect size), and d ≥ 0.8 (large effect size). This statistical approach effectively controlled for Type I error accumulation while ensuring the reliability of the results. Following Bonferroni correction, some comparisons that initially appeared significant no longer met the adjusted thresholds, reflecting the conservative and rigorous approach of this analysis. The presence of trends, such as higher deviations in denser bone types, was explored further through effect size evaluation, providing additional insights into the observed patterns.

### Ethical considerations

This study was conducted in accordance with the ethical standards of the institutional review board (IRB) at Ruijin Hospital, Shanghai Jiao Tong University School of Medicine (Approval Number: 2023439) and the 1964 Helsinki Declaration and its later amendments. All patients provided written informed consent before participation. To ensure patient confidentiality, all data were anonymized during collection and analysis. No identifiable patient information was disclosed.

## Results

### Accuracy analysis between the maxilla and mandible

Implants placed in the mandible exhibited significantly greater deviations compared to the maxilla across all three metrics: coronal deviation (mandible: 1.01 mm ± 0.40mm, maxilla: 0.69 mm ± 0.38 mm, *p* < 0.05), apical deviation (mandible: 1.15 mm ± 0.41mm, maxilla: 0.82 mm ± 0.56mm, *p* < 0.05), and angular deviation (mandible: 1.34° ± 0.99°, maxilla: 2.46° ± 1.92°, *p* < 0.05). These findings suggest that the denser bone structure and reduced surgical accessibility of the mandible may contribute to increased inaccuracies (Table 2).

**TABLE 2:** Presents the deviation analysis of robot-assisted implant placement under various conditions, including coronal deviation, apical deviation, and angular deviation. The data is categorized into the following comparisons: jaw type, specific anato mical positions (position, including anterior, premolar, and molar regions of both the maxilla and mandible), implant dia meter, implant length, and bone density.

		Sample	Coronal deviation Mean ± SD			Apical deviation Mean ± SD			Angular deviation Mean ± SD (°)
			Global (mm)	Lateral (mm)	Vertical (mm)	Global (mm)	Lateral (mm)	Vertical (mm)	
Jaw	Upper	28	0.69 ± 0.38	0.45 ± 0.21	−0.38 ± 0.49	0.82 ± 0.56	0.57 ± 0.47	−0.39 ± 0.50	2.46 ± 1.92
	Lower	26	1.01 ± 0.40	0.31 ± 0.16	0.77 ± 0.58	1.15 ± 0.41	0.34 ± 0.19	−0.83 ± 0.65	1.34 ± 0.99
*p-value*			0.005**	0.012*	0.011*	0.018*	0.024*	0.008**	0.009**
Position	Upper anterior	10	0.90 ± 0.46	0.52 ± 0.26	−0.66 ± 0.50	1.06 ± 0.83	0.71 ± 0.74	−0.69 ± 0.53	3.21 ± 2.22
	Upper premolar	9	0.47 ± 0.13	0.37 ± 0.18	−0.83 ± 0.33	0.59 ± 0.32	0.49 ± 0.32	−0.09 ± 0.33	2.21 ± 1.72
	Upper molar	9	0.71 ± 0.38	0.45 ± 0.16	−0.41 ± 0.47	0.82 ± 0.32	0.53 ± 0.22	−0.40 ± 0.46	1.97 ± 1.78
	Lower anterior	9	1.05 ± 0.36☆	0.39 ± 0.15	−0.76 ± 0.76	1.19 ± 0.41	0.38 ± 0.23	−0.71 ± 0.75	1.57 ± 0.92
	Lower premolar	8	0.88 ± 0.34	0.25 ± 0.18	−0.87 ± 0.33☆	1.11 ± 1.16	0.27 ± 0.21	−1.18 ± 0.39☆	0.77 ± 0.52
	Lower molar	9	1.09 ± 0.51☆	0.30 ± 0.12	−0.69 ± 0.63	1.16 ± 0.59	0.38 ± 0.14	−0.69 ± 0.69	1.68 ± 1.22
*p-value*			0.014*	0.046*	0.035*	0.094	0.201	0.004**	0.039*
Implant diameter	3.3	24	0.84 ± 0.42	0.42 ± 0.20	−0.55 ± 0.62	1.00 ± 0.62	0.54 ± 0.51	−0.55 ± 0.62	2.49 ± 1.82
	4.1	18	0.78 ± 0.42	0.30 ± 0.18	−0.51 ± 0.56	0.89 ± 0.42	0.31 ± 0.17	−0.67 ± 0.70	1.01 ± 1.04★
	4.8	12	0.97 ± 0.41	0.41 ± 0.18	−0.72 ± 0.46	1.11 ± 0.40	0.53 ± 0.19	−0.66 ± 0.51	1.93 ± 1.49
*p-value*			0.506	0.125	0.606	0.529	0.118	0.816	0.019*
Implant length	8	7	0.64 ± 0.13	0.45 ± 0.19	−0.36 ± 0.37	0.72 ± 0.24	0.48 ± 0.28	−0.34 ± 0.36	2.15 ± 1.91
	10	19	0.88 ± 0.53	0.36 ± 0.19	−0.55 ± 0.59	0.94 ± 0.53	0.40 ± 0.14	−0.52 ± 0.61	1.67 ± 1.08
	12	28	0.88 ± 0.38	0.38 ± 0.20	−0.65 ± 0.59	1.08 ± 0.54	0.50 ± 0.49	−0.75 ± 0.65	1.99 ± 1.87
*p-value*			0.397	0.584	0.481	0.225	0.673	0.208	0.74
Bone density	D1	9	1.07 ± 0.38	0.51 ± 0.20	−0.89 ± 0.46	1.06 ± 0.50	0.31 ± 0.19	−0.85 ± 0.47	2.06 ± 1.07
	D2	23	0.88 ± 0.43	0.33 ± 0.21	−0.57 ± 0.62	1.11 ± 0.59	0.54 ± 0.49	−0.74 ± 0.71	1.77 ± 1.74
	D3	13	0.79 ± 0.45	0.39 ± 0.14	−0.84 ± 0.57	0.84 ± 0.43	0.35 ± 0.18	−0.38 ± 0.55	1.72 ± 1.12
	D4	9	0.62 ± 0.31	0.37 ± 0.19	−0.41 ± 0.45	0.81 ± 0.39	0.56 ± 0.33	−0.40 ± 0.44	2.34 ± 2.36
			0.154	0.16	0.274	0.322	0.253	0.169	0.798

* indicates statistically significant differences with ANOVA (p < 0.05), and ** indicates statistically significant differences with ANOVA (p < 0.01).

The Bonferroni correction results use ☆ to indicate a significant difference compared to the Upper premolar group, and ★ to indicate a significant difference compared to the Implant diameter 3.3 group.

### Regional accuracy within the maxilla and mandible

Significant regional variations were observed (Table 2). In the maxilla, the anterior region exhibited higher angular deviations (3.21° ± 2.22°) compared to the premolar and molar regions (*p* < 0.05). In the mandible, the anterior region showed the greatest overall positional deviations (1.19 mm ± 0.41 mm, *p* < 0.05), likely due to restricted access and variable anatomical constraints. These results underscore the importance of region-specific considerations during preoperative planning.

### Accuracy analysis of robotic-assisted implant placement in relation to implant diameter

The analysis of implant diameter revealed no statistically significant differences in accuracy metrics for implants of 3.3 mm, 4.1 mm, and 4.8 mm diameters in most parameters (*p* > 0.05) (Table 2). However, global deviation demonstrated a statistically significant difference, with a p-value of 0.019. Implants with larger diameters (4.8 mm) tended to exhibit slightly higher global deviations compared to smaller diameters. This finding may be attributable to the increased surgical complexity associated with larger implants, which require more extensive bone preparation and stabilization (Pérez-Pevida et al., 2020). Although the effect size is modest, these results suggest that implant diameter should be considered in relation to the patient’s specific anatomical conditions to achieve optimal accuracy.

### Influence of implant length on the accuracy of robotic-assisted implant placement

Implants of varying lengths (8 mm, 10 mm, and 12 mm) were analyzed to assess their impact on placement accuracy. Statistical analysis revealed no significant differences across all evaluated metrics (*p* > 0.05). These findings suggest that implant length, within the tested range, does not substantially affect precision (Table 2). A similar conclusion was reached in a related study, which demonstrated that the shape and length of implants did not influence the precision of implant positioning during robot-assisted immediate implant placement *in vitro* (Wang Y. et al., 2024). Future studies incorporating longer or shorter implants, as well as diverse bone densities, will be necessary to confirm these findings comprehensively.

### Impact of jawbone density on the accuracy of implant placement

**FIGURE 5:**
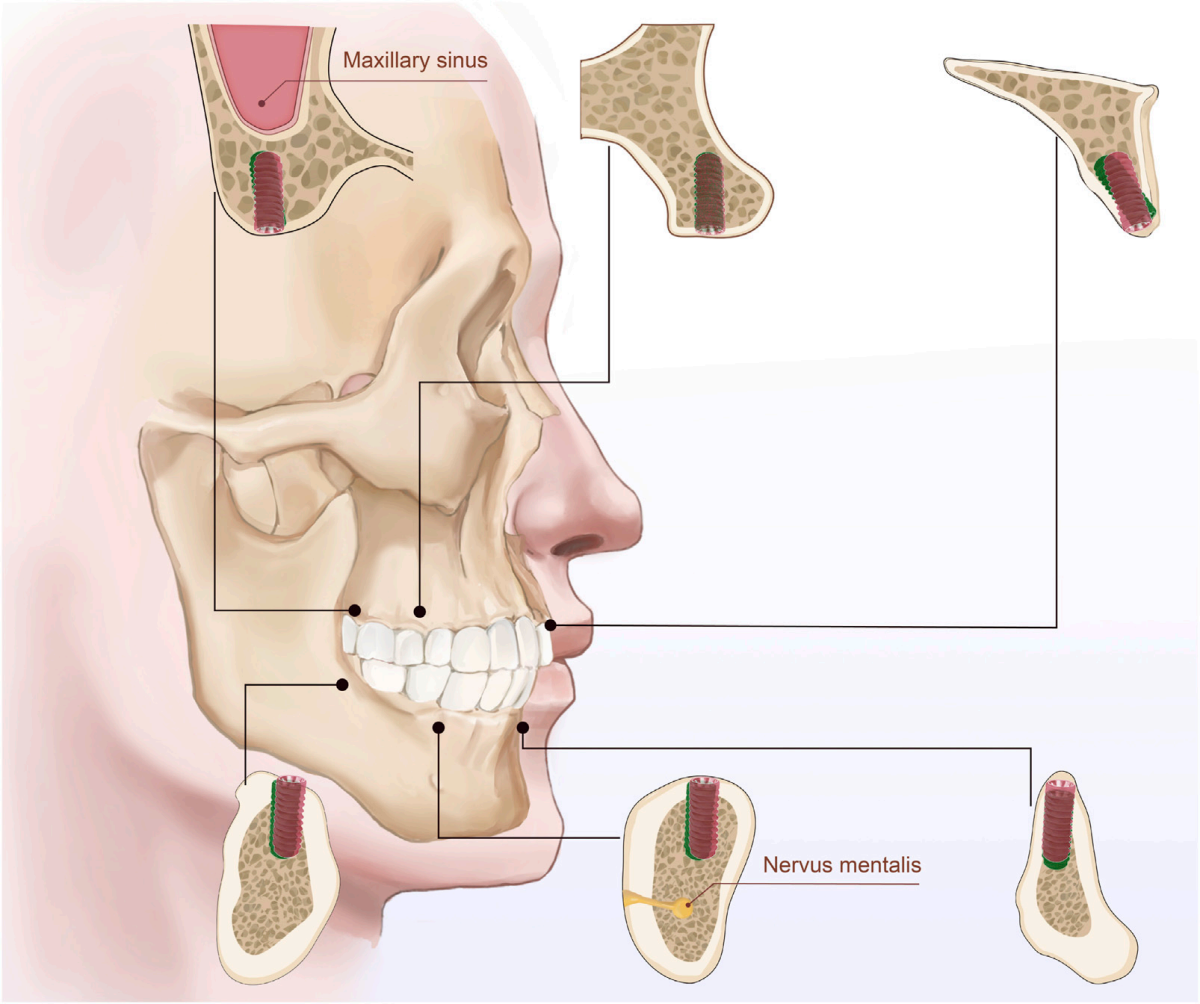
The illustration depicts the bone quality in six different regions of the maxilla and mandible (anterior, premolar, and molar regions) and bone density classified according to Misch’s classification system (D1, D2, D3, and D4). Between the planned implant position (green) and the drilled position (red). The figure emphasizes the bone characteristics in each region and the relationship between implants and surrounding anatomical structures.

The impact of bone density, classified according to Misch’s system (D1–D4), on implant accuracy was assessed. Results indicated no statistically significant differences in accuracy metrics across the four bone density categories (*p* > 0.05) (Table 2). However, a trend was observed where implants placed in D1 bone (the densest bone type) exhibited slightly higher overall deviations, potentially due to the increased resistance encountered during implant insertion, which could affect precision. In contrast, implants placed in D4 bone (the least dense bone type) showed lower vertical deviations, possibly attributed to the ease of achieving alignment in softer bone. Although these trends were not statistically significant, effect size analysis revealed clinically meaningful differences (Figure 5).

For coronal deviation (Table 3), the largest effect size (d = 5.19) was observed between the D1 (highest density) and D4 (lowest density) groups, indicating a significant impact of bone density on coronal positioning precision and suggesting that higher bone density may increase the difficulty of controlling drilling deviations. A decreasing trend in effect size with lower bone density implies that deviations might be more controllable in less dense bone. For apical deviation, significant global effect sizes were noted between D2 and D4 (d = 3.02), and lateral deviations between D3 and D4 (d = 3.74), while vertical deviations in the D1-D3 groups showed notable effect sizes (d = 4.05), highlighting the multidimensional influence of bone density on apical positioning. For angular deviation, moderate to large effect sizes (d = 0.61–1.62) were observed across most groups, with the largest effect size between D2 and D4 (d = 1.62), emphasizing the role of bone density in angular control.

**TABLE 3:** Effect sizes were evaluated using Cohen’s d, d < 0.2: Small effect size. 0.5 ≤ d < 0.5: Small to medium effect size. 0.5 ≤ d < 0.8: Medium effect size. d ≥ 0.8: Large effect size.

	Coronal deviation			Apical deviation			Angular deviation
	Global	Lateral	Vertical	Global	Lateral	Vertical	
D1-D2	2.49	4.75	3.01	0.48	2.92	0.92	1.00
D1-D3	2.96	3.22	0.42	2.14	0.97	4.05	1.38
D1-D4	5.19	2.87	4.22	2.23	3.71	3.95	0.61
D2-D3	1.20	1.86	2.61	2.92	2.71	3.19	0.19
D2-D4	3.55	1.07	1.51	3.02	0.24	2.87	1.62
D3-D4	1.90	0.55	3.66	0.32	3.74	0.18	1.61

## Discussion

The integration of robotic-assisted dental implantation into clinical practice has rapidly gained momentum in recent years, offering the potential to significantly enhance surgical precision, consistency, and predictability (van Riet et al., 2021). These systems provide unique advantages, including real-time feedback, precise control, and the reduction of human error, collectively leading to improved patient outcomes (Isufi et al., 2024). The findings from our study demonstrated remarkable implant placement accuracy, with a mean global platform deviation of 0.76 ± 0.36 mm, apex deviation of 0.85 ± 0.48 mm, and angular deviation of 2.05° ± 1.33°. Furthermore, vertical and horizontal deviations at the platform level were 0.39 ± 0.30 mm and 0.61 ± 0.39 mm, respectively, while the corresponding deviations at the apex level were 0.40 ± 0.36 mm and 0.71 ± 0.39 mm. These values align with or exceed those reported in previous studies (Bolding and Reebye, 2022) which described a passive robotic system achieving a global platform deviation of 1.04 mm, an apex deviation of 0.95 mm, and an angular deviation of 2.56°. Notably, even in edentulous cases, the robotic system maintained its precision despite the anatomical challenges, highlighting its potential for broader clinical applications in complex scenarios. Similarly, Yang et al. (2024), reported comparable accuracy metrics, confirming the exceptional precision of robotic systems in dental implantation. While the results underline the significant potential of robotic systems, the precision of implant placement across different anatomical regions remains underexplored. Complex areas, such as the maxillary anterior aesthetic zone and mandibular posterior region, pose unique challenges due to their intricate bone morphology, high bone density, and proximity to critical anatomical structures (Huang et al., 2024). Our study found that larger-diameter implants exhibited slightly higher deviations, likely due to increased surgical complexity and the limitations of robotic systems in adapting to variations in implant diameter. Conical implants demonstrated greater deviations than cylindrical implants, potentially attributed to their self-tapping design, which requires additional downward force during placement, increasing the risk of misalignment (Ochi et al., 2013). In contrast, cylindrical implants, which lack self-tapping properties, exhibited greater stability. Additionally, larger-diameter implants faced higher resistance in cortical bone regions, and the robotic system’s limited capacity to adapt to these mechanical variations likely contributed to increased deviations (Ozan et al., 2011).

Bone-related factors, such as density, width, and cortical bone thickness, also significantly influence implant placement accuracy. Previous studies (Putra et al., 2020) have demonstrated that poor bone conditions, including low bone density, narrow bone width, and thin cortical bone, increase the risk of deviations during implantation. In line with these findings, our study observed a significant association between bone density and implant accuracy. Denser bone regions, such as the posterior mandible, presented greater resistance during drilling, increasing angular deviations, while softer bone regions, such as the anterior maxilla, posed challenges for achieving primary stability and alignment (Huang et al., 2024). Although no statistically significant association was observed between bone density categories and implant deviation (*p* > 0.05), effect size analysis revealed clinically meaningful differences. For example, the largest coronal deviation effect size (d = 5.19) was observed between D1 (highest density) and D4 (lowest density) bone types, suggesting that higher bone density may increase drilling complexity and deviation risks. Similarly, significant apical and angular deviation effect sizes (d = 3.02–4.05) were observed between various bone density groups, highlighting the multidimensional impact of bone density on implant accuracy. These findings emphasize the need for optimized surgical protocols tailored to bone quality variations to minimize deviations.

These precision differences have profound clinical implications. Robotic-assisted systems have shown significant improvements in accuracy and consistency compared to traditional methods (Chen et al., 2024). However, their application in complex anatomical regions still requires optimization. For high-density cortical bone regions, specially designed pilot drills can be used to reduce slippage during initial positioning. Slowing the drilling speed in the mandibular molar region and incorporating real-time feedback mechanisms can further improve precision. Ensuring strict calibration and registration protocols is critical to maintain navigation accuracy, and systematic training for clinicians can maximize the effectiveness of robotic systems. Collectively, these measures help reduce deviations and improve the initial stability of implants. Despite their potential, robotic systems still face critical challenges. High-quality CBCT imaging and precise calibration remain essential for surgical accuracy, making them sensitive to imaging artifacts and errors during marker registration (Brief et al., 2005; Dong et al., 2012). Reference markers may occasionally become invisible during surgery due to interference from the surgeon or instruments, especially in systems with suboptimal camera designs. However, the robotic system in our study, with dual cameras positioned near the patient’s head, demonstrated improved marker detection, reducing interruptions. Anatomically complex or hard-to-reach implant sites pose additional challenges, as robots may struggle with posture adjustment, increasing operational difficulty. Nevertheless, the system’s follow-up functionality minimizes positional deviations caused by unintended head movements during the procedure.

Furthermore, robotic systems are still in their developmental stages and cannot independently perform complex procedures, such as sinus lifting or advanced bone grafting, which require precise manipulation in anatomically constrained environments. These procedures still rely heavily on the expertise of surgeons for optimal outcomes. While this study focused on implant placement accuracy, it did not address more complex surgical techniques. Future advancements should prioritize introducing tactile feedback, optimizing surgical path planning, and improving robotic posture adjustment for these procedures. Enhancing surgeon-robot interaction could further expand the system’s applicability and safety in challenging scenarios.

This study has several limitations. This study excluded patients with systemic conditions such as diabetes and osteoporosis to ensure a homogeneous study population, thereby enhancing the internal validity of the findings and enabling a more precise assessment of the performance of robot-assisted systems in healthy individuals. However, this exclusion criterion limits the external validity and generalizability of the results, as it does not account for the system’s performance in more complex clinical scenarios. For instance, diabetic patients may experience impaired wound healing, which could compromise the long-term stability of dental implants, while osteoporotic patients may present with reduced bone density, potentially affecting surgical accuracy. Future studies should incorporate a more diverse patient population, including individuals with systemic diseases, to comprehensively evaluate the efficacy and applicability of robotic systems across a broader range of clinical conditions. It was a single-center retrospective study with a limited sample size, necessitating large-scale, multicenter, randomized controlled trials to validate the system’s accuracy and performance. The short-term evaluation of postoperative implant accuracy also leaves questions about long-term success and survival rates unanswered. We plan to conduct follow-up studies to assess long-term outcomes, including osseointegration, mechanical stability, and peri-implant biological changes, to comprehensively evaluate the clinical efficacy of robotic-assisted implantation.

In conclusion, robotic-assisted dental implantation represents a groundbreaking innovation with the potential to revolutionize implant dentistry. Our findings emphasize the system’s ability to achieve exceptional precision across diverse scenarios. Addressing current limitations through technological advancements, standardized protocols, and long-term evaluations will pave the way for robotic systems to become a transformative tool in managing complex implant cases, ultimately setting new standards in dental care. Furthermore, regarding the integration of artificial intelligence (AI) predictive models into the robotic workflow, although this study did not address such technologies, we believe this direction holds great promise. AI predictive models can analyze patient-specific parameters, such as bone density, bone morphology, and other preoperative data, providing personalized assistance for complex case planning and improving procedural success rates. However, the practical integration of AI tools into clinical workflows still faces significant challenges, including data standardization, model validation, and ensuring applicability across diverse clinical environments. Therefore, we recommend future research to explore the feasibility of integrating AI into real-world robotic workflows and to evaluate its potential impact on improving surgical planning efficiency and procedural accuracy. Optimizing AI applications within robotic systems could further advance personalized medicine and open new pathways for managing complex cases. Addressing these technical and clinical challenges will pave the way for the broader adoption of combined robotic and AI technologies in dental implant surgery, ultimately offering patients more precise and effective treatment options.

## Data Availability

The original contributions presented in the study are included in the article/supplementary material, further inquiries can be directed to the corresponding authors.

## References

[B1] Bahrami R. Pourhajibagher M. Nikparto N. Bahador A. (2024). Robot-assisted dental implant surgery procedure: a literature review. J. Dent. Sci. 19 (3), 1359–1368. 10.1016/j.jds.2024.03.011 39035318 PMC11259664

[B2] Block M. S. Emery R. W. (2016). Static or dynamic navigation for implant placement-choosing the method of guidance. J. Oral Maxillofac. Surg. 74 (2), 269–277. 10.1016/j.joms.2015.09.022 26452429

[B3] Bolding S. L. Reebye U. N. (2022). Accuracy of haptic robotic guidance of dental implant surgery for completely edentulous arches. J. Prosthet. Dent. 128 (4), 639–647. 10.1016/j.prosdent.2020.12.048 33678441

[B4] Brief J. Edinger D. Hassfeld S. Eggers G. (2005). Accuracy of image-guided implantology. Clin. Oral Implants Res. 16 (4), 495–501. 10.1111/j.1600-0501.2005.01133.x 16117776

[B5] Chen J. Zhuang M. Tao B. Wu Y. Ye L. Wang F. (2024). Accuracy of immediate dental implant placement with task-autonomous robotic system and navigation system: an *in vitro* study. Clin. Oral Implants Res. 35 (8), 973–983. 10.1111/clr.14104 37248610

[B6] de Almeida E. O. Pellizzer E. P. Goiatto M. C. Margonar R. Rocha E. P. Freitas A. C. Jr. (2010). Computer-guided surgery in implantology: review of basic concepts. J. Craniofac Surg. 21 (6), 1917–1921. 10.1097/SCS.0b013e3181f4b1a0 21119455

[B7] D'Haese J. Ackhurst J. Wismeijer D. De Bruyn H. Tahmaseb A. (2017). Current state of the art of computer-guided implant surgery. Periodontol 73 (1), 121–133. 10.1111/prd.12175 28000275

[B8] Dong X. Niu T. Jia X. Zhu L. (2012). Relationship between x-ray illumination field size and flat field intensity and its impacts on x-ray imaging. Med. Phys. 39 (10), 5901–5909. 10.1118/1.4750054 23039629 PMC3461050

[B9] Howashi M. Tsukiyama Y. Ayukawa Y. Isoda-Akizuki K. Kihara M. Imai Y. (2016). Relationship between the CT value and cortical bone thickness at implant recipient sites and primary implant stability with comparison of different implant types. Clin. Implant Dent. Relat. Res. 18 (1), 107–116. 10.1111/cid.12261 25181581

[B10] Hu Y. Xu S. Li B. Inscoe C. R. Tyndall D. A. Lee Y. Z. (2024). Improving the accuracy of bone mineral density using a multisource CBCT. Sci. Rep. 14 (1), 3887. 10.1038/s41598-024-54529-4 38366012 PMC10873385

[B11] Huang J. Bao J. Tan Z. Shen S. Yu H. (2024). Development and validation of a collaborative robotic platform based on monocular vision for oral surgery: an *in vitro* study. Int. J. Comput. Assist. Radiol. Surg. 19 (9), 1797–1808. 10.1007/s11548-024-03161-8 38822980

[B12] Isufi A. Hsu T. Y. Chogle S. (2024). Robot-assisted and haptic-guided endodontic surgery: a case report. J. Endod. 50 (4), 533–539.e1. 10.1016/j.joen.2024.01.012 38280513

[B13] Jorba-García A. González-Barnadas A. Camps-Font O. Figueiredo R. Valmaseda-Castellón E. (2021). Accuracy assessment of dynamic computer-aided implant placement: a systematic review and meta-analysis. Clin. Oral Investig. 25 (5), 2479–2494. 10.1007/s00784-021-03833-8 33635397

[B14] Misch C. E. (1989). Bone classification, training keys to implant success. Dent. Today 8 (4), 39–44. 2597401

[B15] Misch C. E. (1990). Density of bone: effect on treatment plans, surgical approach, healing, and progressive boen loading. Int. J. Oral Implantol. 6 (2), 23–31. 2073394

[B16] Ochi M. Kanazawa M. Sato D. Kasugai S. Hirano S. Minakuchi S. (2013). Factors affecting accuracy of implant placement with mucosa-supported stereolithographic surgical guides in edentulous mandibles. Comput. Biol. Med. 43 (11), 1653–1660. 10.1016/j.compbiomed.2013.07.029 24209910

[B17] Ozan O. Orhan K. Turkyilmaz I. (2011). Correlation between bone density and angular deviation of implants placed using CT-generated surgical guides. J. Craniofac Surg. 22 (5), 1755–1761. 10.1097/SCS.0b013e31822e6305 21959426

[B18] Pérez-Pevida E. Cherro R. Camps-Font O. Piqué N. (2020). Effects of drilling protocol and bone density on the stability of implants according to different macrogeometries of the implant used: results of an *in vitro* study. Int. J. Oral Maxillofac. Implants 35 (5), 955–964. 10.11607/jomi.8176 32991646

[B19] Putra R. H. Yoda N. Iikubo M. Kataoka Y. Yamauchi K. Koyama S. (2020). Influence of bone condition on implant placement accuracy with computer-guided surgery. Int. J. Implant Dent. 6 (1), 62. 10.1186/s40729-020-00249-z 32951152 PMC7502099

[B20] Shi J. Y. Liu B. L. Wu X. Y. Liu M. Zhang Q. Lai H. C. (2024). Improved positional accuracy of dental implant placement using a haptic and machine-vision-controlled collaborative surgery robot: a pilot randomized controlled trial. J. Clin. Periodontol. 51 (1), 24–32. 10.1111/jcpe.13893 37872750

[B21] Takács A. Hardi E. Cavalcante B. G. N. Szabó B. Kispélyi B. Joób-Fancsaly Á. (2023). Advancing accuracy in guided implant placement: a comprehensive meta-analysis. J. Dent. 139, 104748. 10.1016/j.jdent.2023.104748 37863173

[B22] van Riet T. C. T. Chin Jen Sem K. T. H. Ho J. T. F. Spijker R. Kober J. de Lange J. (2021). Robot technology in dentistry, part one of a systematic review: literature characteristics. Dent. Mater 37 (8), 1217–1226. 10.1016/j.dental.2021.06.001 34158195

[B23] Visconti M. A. Verner F. S. Assis N. M. Devito K. L. (2013). Influence of maxillomandibular positioning in cone beam computed tomography for implant planning. Int. J. Oral Maxillofac. Surg. 42 (7), 880–886. 10.1016/j.ijom.2013.03.001 23566433

[B24] Wang M. Liu F. Zhao X. Wu Y. (2024a). Robot-assisted surgery for dental implant placement: a narrative review. J. Dent. 146, 105034. 10.1016/j.jdent.2024.105034 38729287

[B25] Wang Y. Yu S. Wang Y. Feng Y. Yan Q. Zhang Y. (2024b). Effect of implant shape and length on the accuracy of robot-assisted immediate implant surgery: an *in vitro* study. Clin. Oral Implants Res. 35 (3), 350–357. 10.1111/clr.14232 38174662

[B26] Wu X. Y. Shi J. Y. Qiao S. C. Tonetti M. S. Lai H. C. (2024). Accuracy of robotic surgery for dental implant placement: a systematic review and meta-analysis. Clin. Oral Implants Res. 35 (6), 598–608. 10.1111/clr.14255 38517053

[B27] Yang F. Chen J. Cao R. Tang Q. Liu H. Zheng Y. (2024). Comparative analysis of dental implant placement accuracy: semi-active robotic versus free-hand techniques: a randomized controlled clinical trial. Clin. Implant Dent. Relat. Res. 26 (6), 1149–1161. 10.1111/cid.13375 39161058 PMC11660539

